# 2-(4,5-Dimeth­oxy-2-nitro­phen­yl)-4-meth­oxy-9-phenyl­sulfonyl-9*H*-carbazole-3-carbaldehyde

**DOI:** 10.1107/S1600536814005133

**Published:** 2014-03-12

**Authors:** P. Narayanan, K. Sethusankar, Velu Saravanan, Arasambattu K. Mohanakrishnan

**Affiliations:** a Department of Physics, RKM Vivekananda College (Autonomous), Chennai 600 004, India; b Department of Organic Chemistry, University of Madras, Maraimalai Campus, Chennai 600 025, India

## Abstract

In the title compound, C_28_H_22_N_2_O_8_S, the carbazole ring system is roughly planar, with a maximum deviation of 0.084 (3) Å for the C atom connected to the 4,5-dimeth­oxy-2-nitro­phenyl ring. The dihedral angle between the carbazole system and the dimeth­oxy-substituted nitro­phenyl ring is 57.05 (10)°. The aldehyde C atom deviates by 0.164 (5) Å from its attached carbazole ring system. The mol­ecular structure is stabilized by C—H⋯O inter­actions which generate two *S*(6) and one *S*(7) ring motif. In the crystal, mol­ecules are linked by C—H⋯O hydrogen bonds, forming *R*_3_^3^(15) ring motifs, which are further crosslinked by *R*_3_^2^(19) ring motifs, resulting in (002) layers. The crystal packing also features C—H⋯π inter­actions.

## Related literature   

For the biological activities and uses of carbazole derivatives, see: Itoigawa *et al.* (2000 ▶); Ramsewak *et al.* (1999 ▶). For electronic properties and applications of carbazole derivatives, see: Friend *et al.* (1999 ▶); Zhang *et al.* (2004 ▶). For related structures, see: Gopinath *et al.* (2013 ▶); Narayanan *et al.* (2014 ▶). For the Thorpe–Ingold effect, see: Bassindale (1984 ▶). For standard bond lengths, see: Allen *et al.* (1987 ▶). For graph-set notation: Bernstein *et al.* (1995 ▶).
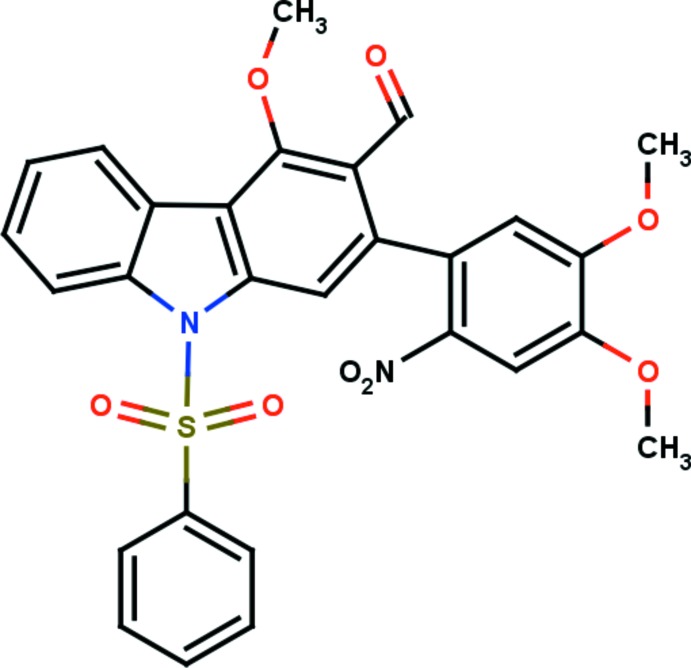


## Experimental   

### 

#### Crystal data  

C_28_H_22_N_2_O_8_S
*M* *_r_* = 546.55
Orthorhombic, 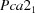

*a* = 8.3976 (6) Å
*b* = 13.7584 (9) Å
*c* = 21.7971 (12) Å
*V* = 2518.4 (3) Å^3^
*Z* = 4
Mo *K*α radiation
μ = 0.19 mm^−1^
*T* = 295 K
0.25 × 0.25 × 0.20 mm

#### Data collection  

Bruker Kappa APEXII CCD diffractometer
Absorption correction: multi-scan (*SADABS*; Bruker, 2008 ▶) *T*_min_ = 0.949, *T*_max_ = 0.971
16717 measured reflections
5538 independent reflections
4047 reflections with *I* > 2σ(*I*)
*R*_int_ = 0.030

#### Refinement  

*R*[*F* ^2^ > 2σ(*F* ^2^)] = 0.043
*wR*(*F* ^2^) = 0.115
*S* = 1.01
5538 reflections
355 parameters
2 restraints
H-atom parameters constrained
Δρ_max_ = 0.37 e Å^−3^
Δρ_min_ = −0.35 e Å^−3^
Absolute structure: Flack (1983 ▶)
Absolute structure parameter: 0.02 (8)

### 

Data collection: *APEX2* (Bruker, 2008 ▶); cell refinement: *APEX2*; data reduction: *SAINT* (Bruker, 2008 ▶); program(s) used to solve structure: *SHELXS97* (Sheldrick, 2008 ▶); program(s) used to refine structure: *SHELXL97* (Sheldrick, 2008 ▶); molecular graphics: *ORTEP–3 for Windows* (Farrugia, 2012 ▶) and *Mercury* (Macrae *et al.*, 2008 ▶); software used to prepare material for publication: *SHELXL97* and *PLATON* (Spek, 2009 ▶).

## Supplementary Material

Crystal structure: contains datablock(s) global, I. DOI: 10.1107/S1600536814005133/rk2423sup1.cif

Structure factors: contains datablock(s) I. DOI: 10.1107/S1600536814005133/rk2423Isup2.hkl

Supporting information file. DOI: 10.1107/S1600536814005133/rk2423Isup3.cml

CCDC reference: 990378

Additional supporting information:  crystallographic information; 3D view; checkCIF report

## Figures and Tables

**Table 1:** Hydrogen-bond geometry (Å, °)

*D*—H⋯*A*	*D*—H	H⋯*A*	*D*⋯*A*	*D*—H⋯*A*
C2—H2⋯O1	0.93	2.35	2.942 (5)	122
C11—H11⋯O2	0.93	2.32	2.927 (3)	122
C25—H25*B*⋯O8	0.96	2.60	3.194 (5)	120
C17—H17⋯O7^i^	0.93	2.56	3.384 (3)	148
C27—H27*B*⋯O3^ii^	0.96	2.56	3.239 (5)	128
C27—H27*B*⋯O8^iii^	0.96	2.58	2.943 (5)	103
C25—H25*A*⋯*Cg*1^iv^	0.96	2.97	3.675 (4)	131

Symmetry codes: (i) 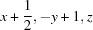; (ii) 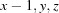; (iii) 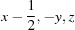; (iv) 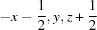.

**Fig. 1.**
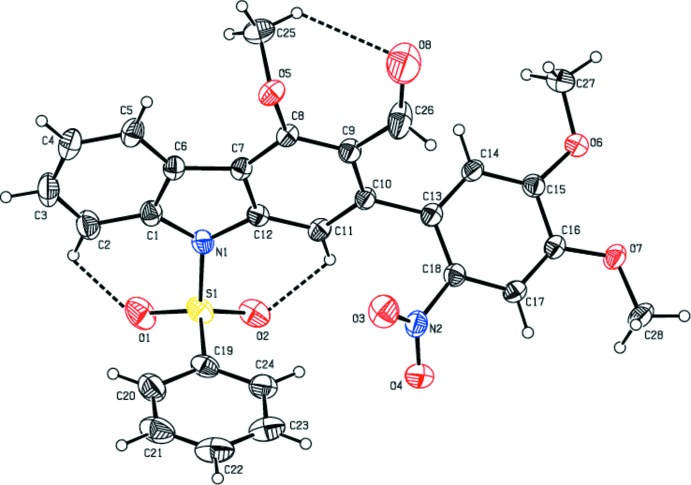
The molecular structure of the title compound with the atom numbering scheme. Displacement ellipsoids are drawn at 30% probability level. H atoms are present as small spheres of arbitary radius. The intramolecular non–classical C—H···O hydrogen bonds, which are generate S(6) and S(7) ring motifs, shown as a dashed lines.

**Fig. 2.**
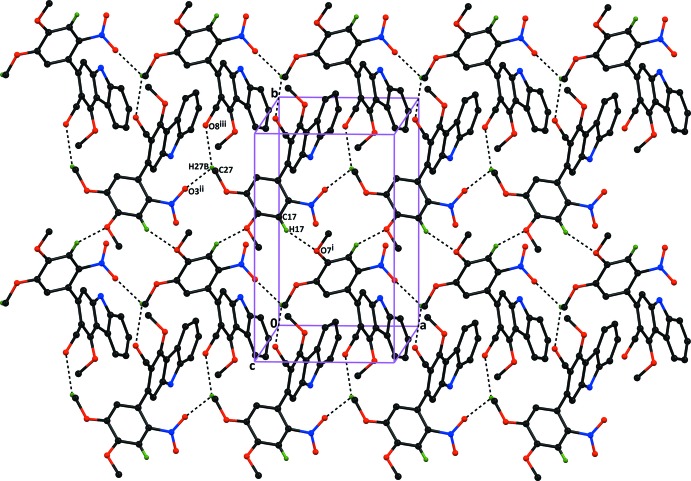
The packing arrangement of the title compound viewed down b axis. The dashed lines indicate C—H···O intermolecular interactions, which are results in R33(15) and R32(19) ring motifs. The hydrogen atoms not involved in the hydrogen bonding and phenylsulfonyl group have been excluded for clarity. Symmetry codes: (i) 1/2+x, 1-y, z; (ii) -1+x, y, z; (iii). -1/2+x, -y, z.

## supplementary crystallographic information

### 1. Comment

Carbazole and its derivative have become quite attractive compounds owing to their applications in pharmacy and molecular electronics. It has been reported that carbazole derivatives exhibit various biological activities such as antitumor (Itoigawa *et al.*, 2000), anti–inflammatory and antimutagenic (Ramsewak *et al.*, 1999). Carbazole derivatives also exhibit electroactivity and luminenscence and are considered to be potential candidates for electronic applications such as colour displays, organic semiconductors, laser and solar cells (Friend *et al.*, 1999; Zhang *et al.*, 2004).

The title compound, C_28_H_22_N_2_O_8_S, comprises a carbazole ring system which is attached to a phenylsulfonyl ring, a dimethoxy substituted nitrophenyl ring, a methoxy group and a aldehyde group. The carbazole ring system is essentially planar with maximum deviation of 0.084 (3)Å for the carbon (C10) atom connected to 4,5–dimethoxy–2–nitrophenyl ring. The aldehyde group carbon (C26) atom and methoxy group oxygen (O5) atom are deviate from the adjacent carbazole ring by -0.164 (5)Å and -0.017 (2)Å, respectively. The carbazole ring system is almost orthogonal to phenyl ring (C19–C24) attached to sulfonyl group with dihedral angle of 88.31 (13)°. The dihedral angle between the carbazole ring and the dimethoxy substituted nitrophenyl ring (C13–C18) is 57.05 (10)°.

The atom S1 has a distorted tetrahedral configuration. The widening of angle O2═S1═O1 (119.83 (15)°) and narrowing of angle N1—S1—C19 (104.91 (12)°) from the ideal tetrahedral value are attributed to the Thorpe–Ingold effect (Bassindale, 1984). As a result of electron–withdrawing character of the phenylsulfonyl group, the bond lengths N1—C1 = 1.430 (3)Å and N1—C12 = 1.417 (3)Å in the molecule are longer than the mean value of 1.355 (14)Å (Allen *et al.*, 1987). The sum of the bond angles around N1 (356.4°) indicate the sp^2^ hybridization. The oxygen atoms O6 & O7 are deviated by 0.025 (2)Å and -0.0105 (19)Å, respectively from the phenyl ring (C13–C18). The title compound exihibits the structural similarities with the already reported related stuctures (Gopinath *et al.*, 2013; Narayanan *et al.*, 2014).

The molecular structure is stabilized by C2—H2···O1, C11—H11···O2 and C25—H25B···O8 intramolecular interactions, which are generate two S(6) and one S(7) ring motifs (Fig. 1). In the crystal packing, molecules are linked by C17—H17···O7^i^ and C27—H27B···O3^ii^ intermolecular hydrogen bondings form *R*_3_^3^(15) ring motifs and these ring motifs are further cross linked by C27—H27B···O8^iii^ hydrogen bond forms *R*_3_^2^(19) ring motifs, resulting in two dimensional supramolecular networks (Fig. 2) (Bernstein *et al.*, 1995). The crystal packing is also characterized by C25—H25A···*Cg*1^iv^ interactions. The packing view of the title compound is shown in Fig. 2. Symmetry codes: (i) 1/2+*x*, 1-*y*, *z*; (ii) -1+*x*, *y*, *z*; (iii) -1/2+*x*, -*y*, *z*; (vi) -1/2-*x*, *y*,, 1/2+*z*.

### 2. Experimental

A solution of 3–(bromomethyl)–2–(4,5–dimethoxy–2–nitrophenyl)– 4–methoxy–9–(phenylsulfonyl)–9*H*–carbazole (1.53 g, 2.5 mmol) and bis(tetrabutylammonium) dichromate (2.63 g, 3.75 mmol) in dry CHCl_3_ (50 ml) was refluxed for 10 h. The susequent removal of solvent in vacuo followed by column chromatographic (silica gel; hexane–ethyl acetate, 4:1) purification afforded 9–(phenylsulfonyl)–2–(4,5–dimethoxy– 2–nitrophenyl)–4–methoxy–9*H*–carbazole–3–carbaldehyde (1.19 g, 86%) as a colourless solid. Single crystal suitable for X-ray diffraction were prepared by slow evaporation of a solution of the title compound in chloroform (CHCl_3_) at room temperature. M.p. 471–473 K.

### 3. Refinement

The positions of hydrogen atoms were localized from the difference electron density maps and their distances were geometrically constrained. The hydrogen atoms bound to the C atoms are treated as riding atoms, with d(C—H)=0.93 and *U*iso(H) = 1.2Ueq(C) for aromatic and aldehyde group, d(C—H)=0.96 and *U*iso(H) =1.5*U*eq(C) for methyl groups. The rotation angles for methyl groups were optimized by least squares. In the absence of significant anomalous dispersion effects, an absolute structure was not determined and 1310 Friedel pairs were merged.

### Crystal data

**Table:**

C_28_H_22_N_2_O_8_S	*D*_x_ = 1.441 Mg m^−^^3^
*M**_r_* = 546.55	Melting point = 471–473 K
Orthorhombic, *P**c**a*2_1_	Mo *K*α radiation, λ = 0.71073 Å
Hall symbol: P 2c -2ac	Cell parameters from 4047 reflections
*a* = 8.3976 (6) Å	θ = 2.4–29.9°
*b* = 13.7584 (9) Å	µ = 0.19 mm^−^^1^
*c* = 21.7971 (12) Å	*T* = 295 K
*V* = 2518.4 (3) Å^3^	Block, colourless
*Z* = 4	0.25 × 0.25 × 0.20 mm
*F*(000) = 1136	

### Data collection

**Table:**

Bruker Kappa APEX–II CCD diffractometer	5538 independent reflections
Radiation source: fine–focus sealed tube	4047 reflections with *I* > 2σ(*I*)
Graphite monochromator	*R*_int_ = 0.030
ω– & φ–scans	θ_max_ = 29.9°, θ_min_ = 2.4°
Absorption correction: multi-scan (*SADABS*; Bruker, 2008)	*h* = −11→11
*T*_min_ = 0.949, *T*_max_ = 0.971	*k* = −19→12
16717 measured reflections	*l* = −16→30

### Refinement

**Table:**

Refinement on *F*^2^	Secondary atom site location: difference Fourier map
Least-squares matrix: full	Hydrogen site location: inferred from neighbouring sites
*R*[*F*^2^ > 2σ(*F*^2^)] = 0.043	H-atom parameters constrained
*wR*(*F*^2^) = 0.115	*w* = 1/[σ^2^(*F*_o_^2^) + (0.059*P*)^2^ + 0.2808*P*] where *P* = (*F*_o_^2^ + 2*F*_c_^2^)/3
*S* = 1.01	(Δ/σ)_max_ < 0.001
5538 reflections	Δρ_max_ = 0.37 e Å^−^^3^
355 parameters	Δρ_min_ = −0.35 e Å^−^^3^
2 restraints	Absolute structure: Flack (1983)
Primary atom site location: structure-invariant direct methods	Absolute structure parameter: 0.02 (8)

### Special details

**Table:**

Geometry. All s.u.'s (except the s.u. in the dihedral angle between two l.s. planes) are estimated using the full covariance matrix. The cell s.u.'s are taken into account individually in the estimation of s.u.'s in distances, angles and torsion angles; correlations between s.u.'s in cell parameters are only used when they are defined by crystal symmetry. An approximate (isotropic) treatment of cell s.u.'s is used for estimating s.u.'s involving l.s. planes.
Refinement. Refinement of *F*^2^ against ALL reflections. The weighted *R*–factor *wR* and goodness of fit *S* are based on *F*^2^, conventional *R*–factors *R* are based on *F*, with *F* set to zero for negative *F*^2^. The threshold expression of *F*^2^ > σ(*F*^2^) is used only for calculating *R*–factors(gt) *etc*. and is not relevant to the choice of reflections for refinement. *R*–factors based on *F*^2^ are statistically about twice as large as those based on *F*, and *R*–factors based on ALL data will be even larger.

### Fractional atomic coordinates and isotropic or equivalent isotropic displacement parameters (Å^2^)

**Table:**

	*x*	*y*	*z*	*U*_iso_*/*U*_eq_	
C1	0.8650 (3)	0.1054 (2)	0.40810 (13)	0.0448 (6)	
C2	0.9302 (4)	0.0894 (3)	0.35094 (14)	0.0615 (8)	
H2	0.9377	0.1391	0.3221	0.074*	
C3	0.9838 (4)	−0.0031 (3)	0.33812 (16)	0.0708 (10)	
H3	1.0298	−0.0156	0.3001	0.085*	
C4	0.9712 (4)	−0.0772 (3)	0.37992 (15)	0.0598 (8)	
H4	1.0067	−0.1391	0.3694	0.072*	
C5	0.9070 (3)	−0.0617 (2)	0.43698 (14)	0.0511 (7)	
H5	0.8994	−0.1121	0.4652	0.061*	
C6	0.8536 (3)	0.0312 (2)	0.45165 (12)	0.0396 (6)	
C7	0.7830 (3)	0.07281 (18)	0.50642 (12)	0.0366 (5)	
C8	0.7490 (3)	0.03425 (17)	0.56383 (12)	0.0371 (5)	
C9	0.6929 (3)	0.09573 (19)	0.61001 (12)	0.0434 (6)	
C10	0.6676 (3)	0.19546 (18)	0.59770 (12)	0.0382 (5)	
C11	0.6948 (3)	0.23323 (19)	0.53996 (12)	0.0391 (6)	
H11	0.6732	0.2981	0.5313	0.047*	
C12	0.7553 (3)	0.17175 (18)	0.49529 (11)	0.0365 (5)	
C13	0.6053 (3)	0.26215 (18)	0.64630 (12)	0.0387 (5)	
C14	0.4585 (3)	0.24500 (19)	0.67360 (12)	0.0419 (6)	
H14	0.4016	0.1895	0.6630	0.050*	
C15	0.3949 (3)	0.30877 (18)	0.71640 (13)	0.0417 (6)	
C16	0.4779 (3)	0.39378 (19)	0.73222 (11)	0.0393 (5)	
C17	0.6221 (3)	0.41252 (19)	0.70510 (12)	0.0408 (6)	
H17	0.6778	0.4690	0.7146	0.049*	
C18	0.6841 (3)	0.34688 (19)	0.66353 (12)	0.0401 (6)	
C19	1.0216 (3)	0.3359 (2)	0.43564 (14)	0.0496 (7)	
C20	1.1564 (4)	0.3137 (2)	0.4026 (2)	0.0661 (9)	
H20	1.1486	0.2835	0.3646	0.079*	
C21	1.3031 (4)	0.3368 (3)	0.4268 (2)	0.0798 (12)	
H21	1.3949	0.3226	0.4047	0.096*	
C22	1.3149 (4)	0.3804 (3)	0.4830 (2)	0.0792 (12)	
H22	1.4146	0.3946	0.4993	0.095*	
C23	1.1808 (5)	0.4032 (3)	0.5151 (2)	0.0798 (11)	
H23	1.1896	0.4341	0.5529	0.096*	
C24	1.0324 (4)	0.3809 (2)	0.49226 (17)	0.0599 (8)	
H24	0.9410	0.3957	0.5145	0.072*	
C25	0.6625 (4)	−0.1291 (2)	0.55932 (18)	0.0603 (8)	
H25A	0.6311	−0.1181	0.5176	0.090*	
H25B	0.5721	−0.1210	0.5858	0.090*	
H25C	0.7031	−0.1940	0.5634	0.090*	
C26	0.6670 (6)	0.0594 (3)	0.67271 (17)	0.0833 (12)	
H26	0.7082	0.0952	0.7052	0.100*	
C27	0.1723 (4)	0.2068 (2)	0.73733 (17)	0.0636 (9)	
H27A	0.2407	0.1538	0.7485	0.095*	
H27B	0.1418	0.2005	0.6951	0.095*	
H27C	0.0788	0.2058	0.7627	0.095*	
C28	0.4937 (4)	0.5310 (2)	0.79770 (17)	0.0663 (9)	
H28A	0.5144	0.5765	0.7653	0.099*	
H28B	0.5928	0.5081	0.8142	0.099*	
H28C	0.4335	0.5623	0.8295	0.099*	
N1	0.8002 (3)	0.19235 (16)	0.43400 (11)	0.0443 (5)	
N2	0.8427 (3)	0.3687 (2)	0.63994 (11)	0.0508 (6)	
O1	0.8401 (3)	0.2956 (2)	0.34239 (11)	0.0744 (7)	
O2	0.7169 (2)	0.36484 (15)	0.43482 (11)	0.0597 (5)	
O3	0.9392 (3)	0.30255 (18)	0.63549 (13)	0.0732 (7)	
O4	0.8724 (3)	0.45314 (18)	0.62628 (12)	0.0744 (7)	
O5	0.7846 (2)	−0.06046 (13)	0.57617 (9)	0.0473 (5)	
O6	0.2548 (3)	0.29660 (14)	0.74591 (10)	0.0550 (5)	
O7	0.4055 (2)	0.45109 (14)	0.77418 (9)	0.0497 (5)	
O8	0.5939 (8)	−0.0159 (2)	0.68447 (18)	0.168 (2)	
S1	0.83364 (8)	0.30432 (5)	0.40724 (4)	0.05068 (19)	

### Atomic displacement parameters (Å^2^)

**Table:**

	*U*^11^	*U*^22^	*U*^33^	*U*^12^	*U*^13^	*U*^23^
C1	0.0412 (13)	0.0577 (17)	0.0356 (13)	−0.0009 (12)	−0.0036 (11)	−0.0048 (13)
C2	0.0640 (19)	0.079 (2)	0.0416 (17)	−0.0063 (17)	0.0054 (14)	−0.0041 (15)
C3	0.063 (2)	0.102 (3)	0.0476 (19)	0.0011 (19)	0.0092 (15)	−0.0243 (19)
C4	0.0575 (18)	0.069 (2)	0.0532 (19)	0.0106 (15)	−0.0038 (14)	−0.0212 (16)
C5	0.0435 (14)	0.0604 (18)	0.0495 (17)	0.0090 (13)	−0.0059 (12)	−0.0139 (14)
C6	0.0319 (12)	0.0496 (16)	0.0372 (14)	0.0014 (11)	−0.0048 (9)	−0.0059 (11)
C7	0.0321 (11)	0.0412 (13)	0.0367 (13)	0.0015 (10)	−0.0052 (9)	−0.0046 (10)
C8	0.0418 (13)	0.0336 (13)	0.0358 (13)	0.0038 (11)	−0.0059 (10)	−0.0001 (10)
C9	0.0535 (15)	0.0396 (14)	0.0369 (14)	0.0042 (12)	−0.0008 (11)	0.0023 (11)
C10	0.0444 (13)	0.0344 (13)	0.0358 (14)	0.0015 (11)	0.0004 (10)	−0.0023 (10)
C11	0.0442 (13)	0.0333 (13)	0.0397 (14)	0.0042 (11)	−0.0053 (11)	0.0003 (11)
C12	0.0371 (11)	0.0414 (14)	0.0311 (13)	−0.0015 (11)	−0.0049 (9)	0.0038 (10)
C13	0.0473 (14)	0.0354 (13)	0.0334 (13)	0.0037 (11)	−0.0032 (10)	0.0016 (10)
C14	0.0547 (15)	0.0324 (13)	0.0387 (14)	−0.0034 (11)	−0.0003 (11)	−0.0033 (10)
C15	0.0460 (13)	0.0398 (14)	0.0395 (14)	0.0009 (11)	0.0019 (11)	0.0026 (11)
C16	0.0452 (13)	0.0356 (13)	0.0370 (14)	0.0048 (11)	−0.0026 (10)	−0.0033 (10)
C17	0.0459 (13)	0.0328 (12)	0.0437 (15)	0.0005 (11)	−0.0039 (11)	−0.0010 (11)
C18	0.0422 (13)	0.0408 (15)	0.0372 (14)	0.0006 (11)	−0.0010 (10)	−0.0009 (11)
C19	0.0480 (14)	0.0421 (15)	0.0588 (19)	0.0000 (12)	0.0029 (13)	0.0186 (14)
C20	0.0587 (18)	0.068 (2)	0.072 (2)	0.0017 (16)	0.0180 (16)	0.0060 (18)
C21	0.0464 (18)	0.070 (2)	0.123 (4)	−0.0021 (17)	0.020 (2)	0.011 (2)
C22	0.0528 (19)	0.056 (2)	0.129 (4)	−0.0050 (16)	−0.009 (2)	0.013 (2)
C23	0.073 (2)	0.062 (2)	0.104 (3)	−0.0222 (19)	−0.012 (2)	−0.004 (2)
C24	0.0561 (17)	0.0543 (18)	0.069 (2)	−0.0086 (14)	0.0060 (15)	0.0004 (16)
C25	0.0645 (19)	0.0419 (16)	0.075 (2)	−0.0013 (14)	−0.0089 (16)	0.0048 (15)
C26	0.157 (4)	0.0522 (18)	0.0410 (18)	0.0338 (19)	0.011 (2)	0.0073 (16)
C27	0.0669 (19)	0.058 (2)	0.065 (2)	−0.0191 (16)	0.0083 (15)	0.0023 (15)
C28	0.0626 (19)	0.0521 (19)	0.084 (3)	−0.0076 (15)	0.0101 (16)	−0.0333 (17)
N1	0.0502 (12)	0.0490 (13)	0.0336 (12)	−0.0035 (10)	0.0008 (9)	0.0015 (10)
N2	0.0500 (14)	0.0579 (16)	0.0446 (14)	−0.0007 (12)	0.0018 (10)	−0.0125 (11)
O1	0.0811 (17)	0.0983 (19)	0.0439 (14)	−0.0043 (13)	−0.0030 (11)	0.0244 (12)
O2	0.0505 (11)	0.0575 (12)	0.0710 (14)	0.0094 (9)	−0.0002 (10)	0.0204 (11)
O3	0.0489 (12)	0.0819 (17)	0.0890 (19)	0.0147 (12)	0.0014 (12)	−0.0089 (13)
O4	0.0733 (15)	0.0623 (15)	0.0878 (19)	−0.0204 (12)	0.0230 (13)	−0.0121 (13)
O5	0.0556 (11)	0.0359 (10)	0.0504 (12)	0.0089 (8)	−0.0117 (8)	−0.0006 (8)
O6	0.0559 (11)	0.0523 (12)	0.0570 (13)	−0.0130 (10)	0.0151 (9)	−0.0105 (9)
O7	0.0508 (10)	0.0424 (11)	0.0559 (12)	0.0031 (9)	0.0052 (9)	−0.0143 (9)
O8	0.346 (7)	0.068 (2)	0.090 (3)	−0.015 (3)	0.072 (3)	0.0203 (17)
S1	0.0495 (4)	0.0595 (5)	0.0431 (4)	0.0009 (3)	−0.0010 (3)	0.0174 (3)

### Geometric parameters (Å, º)

**Table:**

C1—C2	1.379 (4)	C18—N2	1.459 (4)
C1—C6	1.398 (4)	C19—C20	1.376 (4)
C1—N1	1.430 (3)	C19—C24	1.383 (5)
C2—C3	1.379 (5)	C19—S1	1.751 (3)
C2—H2	0.9300	C20—C21	1.377 (5)
C3—C4	1.371 (5)	C20—H20	0.9300
C3—H3	0.9300	C21—C22	1.368 (7)
C4—C5	1.372 (4)	C21—H21	0.9300
C4—H4	0.9300	C22—C23	1.363 (6)
C5—C6	1.391 (4)	C22—H22	0.9300
C5—H5	0.9300	C23—C24	1.377 (5)
C6—C7	1.451 (3)	C23—H23	0.9300
C7—C8	1.389 (4)	C24—H24	0.9300
C7—C12	1.402 (3)	C25—O5	1.442 (4)
C8—O5	1.364 (3)	C25—H25A	0.9600
C8—C9	1.397 (4)	C25—H25B	0.9600
C9—C10	1.414 (3)	C25—H25C	0.9600
C9—C26	1.471 (4)	C26—O8	1.231 (5)
C10—C11	1.381 (4)	C26—H26	0.9300
C10—C13	1.496 (4)	C27—O6	1.429 (3)
C11—C12	1.386 (4)	C27—H27A	0.9600
C11—H11	0.9300	C27—H27B	0.9600
C12—N1	1.417 (3)	C27—H27C	0.9600
C13—C14	1.388 (4)	C28—O7	1.422 (3)
C13—C18	1.392 (4)	C28—H28A	0.9600
C14—C15	1.388 (4)	C28—H28B	0.9600
C14—H14	0.9300	C28—H28C	0.9600
C15—O6	1.351 (3)	N1—S1	1.671 (2)
C15—C16	1.404 (4)	N2—O3	1.223 (3)
C16—O7	1.352 (3)	N2—O4	1.225 (3)
C16—C17	1.372 (4)	O1—S1	1.420 (2)
C17—C18	1.381 (4)	O2—S1	1.420 (2)
C17—H17	0.9300		
			
C2—C1—C6	121.6 (3)	C20—C19—C24	120.8 (3)
C2—C1—N1	129.9 (3)	C20—C19—S1	120.1 (3)
C6—C1—N1	108.5 (2)	C24—C19—S1	119.0 (2)
C1—C2—C3	117.4 (3)	C19—C20—C21	119.0 (4)
C1—C2—H2	121.3	C19—C20—H20	120.5
C3—C2—H2	121.3	C21—C20—H20	120.5
C4—C3—C2	121.8 (3)	C22—C21—C20	120.6 (4)
C4—C3—H3	119.1	C22—C21—H21	119.7
C2—C3—H3	119.1	C20—C21—H21	119.7
C3—C4—C5	121.1 (3)	C23—C22—C21	120.1 (4)
C3—C4—H4	119.4	C23—C22—H22	120.0
C5—C4—H4	119.4	C21—C22—H22	120.0
C4—C5—C6	118.6 (3)	C22—C23—C24	120.7 (4)
C4—C5—H5	120.7	C22—C23—H23	119.6
C6—C5—H5	120.7	C24—C23—H23	119.6
C5—C6—C1	119.5 (2)	C23—C24—C19	118.8 (3)
C5—C6—C7	133.1 (3)	C23—C24—H24	120.6
C1—C6—C7	107.4 (2)	C19—C24—H24	120.6
C8—C7—C12	119.5 (2)	O5—C25—H25A	109.5
C8—C7—C6	132.4 (2)	O5—C25—H25B	109.5
C12—C7—C6	108.0 (2)	H25A—C25—H25B	109.5
O5—C8—C7	119.9 (2)	O5—C25—H25C	109.5
O5—C8—C9	120.7 (2)	H25A—C25—H25C	109.5
C7—C8—C9	119.2 (2)	H25B—C25—H25C	109.5
C8—C9—C10	120.1 (2)	O8—C26—C9	123.6 (4)
C8—C9—C26	120.9 (2)	O8—C26—H26	118.2
C10—C9—C26	118.9 (2)	C9—C26—H26	118.2
C11—C10—C9	120.9 (2)	O6—C27—H27A	109.5
C11—C10—C13	118.2 (2)	O6—C27—H27B	109.5
C9—C10—C13	120.9 (2)	H27A—C27—H27B	109.5
C10—C11—C12	118.1 (2)	O6—C27—H27C	109.5
C10—C11—H11	120.9	H27A—C27—H27C	109.5
C12—C11—H11	120.9	H27B—C27—H27C	109.5
C11—C12—C7	122.1 (2)	O7—C28—H28A	109.5
C11—C12—N1	129.6 (2)	O7—C28—H28B	109.5
C7—C12—N1	108.3 (2)	H28A—C28—H28B	109.5
C14—C13—C18	116.7 (2)	O7—C28—H28C	109.5
C14—C13—C10	120.6 (2)	H28A—C28—H28C	109.5
C18—C13—C10	122.6 (2)	H28B—C28—H28C	109.5
C15—C14—C13	121.5 (2)	C12—N1—C1	107.8 (2)
C15—C14—H14	119.3	C12—N1—S1	123.94 (18)
C13—C14—H14	119.3	C1—N1—S1	124.68 (19)
O6—C15—C14	125.2 (2)	O3—N2—O4	123.5 (3)
O6—C15—C16	114.7 (2)	O3—N2—C18	118.7 (3)
C14—C15—C16	120.0 (2)	O4—N2—C18	117.9 (2)
O7—C16—C17	125.4 (2)	C8—O5—C25	114.8 (2)
O7—C16—C15	115.4 (2)	C15—O6—C27	117.9 (2)
C17—C16—C15	119.2 (2)	C16—O7—C28	117.4 (2)
C16—C17—C18	119.5 (2)	O1—S1—O2	119.83 (15)
C16—C17—H17	120.3	O1—S1—N1	106.05 (14)
C18—C17—H17	120.3	O2—S1—N1	106.06 (12)
C17—C18—C13	123.1 (2)	O1—S1—C19	109.79 (15)
C17—C18—N2	116.1 (2)	O2—S1—C19	109.09 (15)
C13—C18—N2	120.8 (2)	N1—S1—C19	104.91 (12)
			
C6—C1—C2—C3	0.1 (4)	C15—C16—C17—C18	−1.0 (4)
N1—C1—C2—C3	−179.8 (3)	C16—C17—C18—C13	1.4 (4)
C1—C2—C3—C4	1.0 (5)	C16—C17—C18—N2	−175.9 (2)
C2—C3—C4—C5	−1.3 (5)	C14—C13—C18—C17	−0.6 (4)
C3—C4—C5—C6	0.4 (4)	C10—C13—C18—C17	175.2 (2)
C4—C5—C6—C1	0.6 (4)	C14—C13—C18—N2	176.7 (2)
C4—C5—C6—C7	−179.2 (3)	C10—C13—C18—N2	−7.6 (4)
C2—C1—C6—C5	−0.9 (4)	C24—C19—C20—C21	0.0 (5)
N1—C1—C6—C5	179.0 (2)	S1—C19—C20—C21	−178.1 (3)
C2—C1—C6—C7	178.9 (2)	C19—C20—C21—C22	0.6 (5)
N1—C1—C6—C7	−1.1 (3)	C20—C21—C22—C23	−1.3 (6)
C5—C6—C7—C8	2.3 (4)	C21—C22—C23—C24	1.4 (6)
C1—C6—C7—C8	−177.5 (2)	C22—C23—C24—C19	−0.8 (6)
C5—C6—C7—C12	178.9 (3)	C20—C19—C24—C23	0.1 (5)
C1—C6—C7—C12	−0.9 (3)	S1—C19—C24—C23	178.2 (3)
C12—C7—C8—O5	−176.0 (2)	C8—C9—C26—O8	−48.4 (6)
C6—C7—C8—O5	0.3 (4)	C10—C9—C26—O8	134.7 (4)
C12—C7—C8—C9	−2.1 (3)	C11—C12—N1—C1	176.3 (2)
C6—C7—C8—C9	174.2 (2)	C7—C12—N1—C1	−3.2 (3)
O5—C8—C9—C10	175.3 (2)	C11—C12—N1—S1	16.8 (4)
C7—C8—C9—C10	1.5 (4)	C7—C12—N1—S1	−162.79 (18)
O5—C8—C9—C26	−1.5 (4)	C2—C1—N1—C12	−177.3 (3)
C7—C8—C9—C26	−175.3 (3)	C6—C1—N1—C12	2.7 (3)
C8—C9—C10—C11	1.2 (4)	C2—C1—N1—S1	−18.0 (4)
C26—C9—C10—C11	178.1 (3)	C6—C1—N1—S1	162.05 (18)
C8—C9—C10—C13	179.1 (2)	C17—C18—N2—O3	137.1 (3)
C26—C9—C10—C13	−4.0 (4)	C13—C18—N2—O3	−40.3 (4)
C9—C10—C11—C12	−3.2 (4)	C17—C18—N2—O4	−42.0 (3)
C13—C10—C11—C12	178.8 (2)	C13—C18—N2—O4	140.5 (3)
C10—C11—C12—C7	2.6 (3)	C7—C8—O5—C25	−86.9 (3)
C10—C11—C12—N1	−176.9 (2)	C9—C8—O5—C25	99.3 (3)
C8—C7—C12—C11	0.1 (4)	C14—C15—O6—C27	6.6 (4)
C6—C7—C12—C11	−177.0 (2)	C16—C15—O6—C27	−173.2 (3)
C8—C7—C12—N1	179.7 (2)	C17—C16—O7—C28	−8.0 (4)
C6—C7—C12—N1	2.6 (3)	C15—C16—O7—C28	171.9 (3)
C11—C10—C13—C14	118.4 (3)	C12—N1—S1—O1	−167.4 (2)
C9—C10—C13—C14	−59.5 (3)	C1—N1—S1—O1	36.5 (3)
C11—C10—C13—C18	−57.2 (3)	C12—N1—S1—O2	−38.9 (2)
C9—C10—C13—C18	124.9 (3)	C1—N1—S1—O2	164.9 (2)
C18—C13—C14—C15	−0.7 (4)	C12—N1—S1—C19	76.5 (2)
C10—C13—C14—C15	−176.5 (2)	C1—N1—S1—C19	−79.7 (2)
C13—C14—C15—O6	−178.7 (2)	C20—C19—S1—O1	−24.5 (3)
C13—C14—C15—C16	1.1 (4)	C24—C19—S1—O1	157.3 (2)
O6—C15—C16—O7	−0.4 (3)	C20—C19—S1—O2	−157.6 (2)
C14—C15—C16—O7	179.8 (2)	C24—C19—S1—O2	24.2 (3)
O6—C15—C16—C17	179.5 (2)	C20—C19—S1—N1	89.1 (3)
C14—C15—C16—C17	−0.3 (4)	C24—C19—S1—N1	−89.1 (3)
O7—C16—C17—C18	178.9 (2)		

### Hydrogen-bond geometry (Å, º)

**Table:**

*D*—H···*A*	*D*—H	H···*A*	*D*···*A*	*D*—H···*A*
C2—H2···O1	0.93	2.35	2.942 (5)	122
C11—H11···O2	0.93	2.32	2.927 (3)	122
C25—H25*B*···O8	0.96	2.60	3.194 (5)	120
C17—H17···O7^i^	0.93	2.56	3.384 (3)	148
C27—H27*B*···O3^ii^	0.96	2.56	3.239 (5)	128
C27—H27*B*···O8^iii^	0.96	2.58	2.943 (5)	103
C25—H25*A*···*Cg*1^iv^	0.96	2.97	3.675 (4)	131

Symmetry codes: (i) *x*+1/2, −*y*+1, *z*; (ii) *x*−1, *y*, *z*; (iii) *x*−1/2, −*y*, *z*; (iv) −*x*−1/2, *y*, *z*+1/2.
